# Lung Protection After Severe Thermal Burns With Adenosine, Lidocaine, and Magnesium (ALM) Resuscitation and Importance of Shams in a Rat Model

**DOI:** 10.1093/jbcr/irad127

**Published:** 2023-08-21

**Authors:** Lisa M Davenport, Hayley L Letson, Geoffrey P Dobson

**Affiliations:** Heart and Trauma Research Laboratory, College of Medicine and Dentistry, James Cook University, Queensland 4811, Australia; Heart and Trauma Research Laboratory, College of Medicine and Dentistry, James Cook University, Queensland 4811, Australia; Heart and Trauma Research Laboratory, College of Medicine and Dentistry, James Cook University, Queensland 4811, Australia

## Abstract

The management of severe burns remains a complex challenge. Adenosine, lidocaine, and magnesium (ALM) resuscitation therapy has been shown to protect against hemorrhagic shock and traumatic injury. The aim of the present study was to investigate the early protective effects of small-volume ALM fluid resuscitation in a rat model of 30% total body surface area (TBSA) thermal injury. Male Sprague–Dawley rats (320–340 g; *n* = 25) were randomly assigned to: 1) Sham (surgical instrumentation and saline infusion, without burn, *n* = 5), 2) Saline resuscitation group (*n* = 10), or 3) ALM resuscitation group (*n* = 10). Treatments were initiated 15-min after burn trauma, including 0.7 mL/kg 3% NaCl ± ALM bolus and 0.25–0.5 mL/kg/h 0.9% NaCl ± ALM drip, with animals monitored to 8.25-hr post-burn. Hemodynamics, cardiac function, blood chemistry, hematology, endothelial injury markers and histopathology were assessed. Survival was 100% for Shams and 90% for both ALM and Saline groups. Shams underwent significant physiological, immune and hematological changes over time as a result of surgical traums. ALM significantly reduced malondialdehyde levels in the lungs compared to Saline (*P* = .023), and showed minimal alveolar destruction and inflammatory cell infiltration (*P* < .001). ALM also improved cardiac function and oxygen delivery (21%, *P* = .418 vs Saline), reduced gut injury (*P* < .001 vs Saline), and increased plasma adiponectin (*P* < .001 vs baseline). Circulating levels of the acute phase protein alpha 1-acid glycoprotein (AGP) increased 1.6-times (*P* < .001), which may have impacted ALM's therapeutic efficacy. We conclude that small-volume ALM therapy significantly reduced lung oxidative stress and preserved alveolar integrity following severe burn trauma. Further studies are required to assess higher ALM doses with longer monitoring periods.

## INTRODUCTION

The acute management of severe burns presents a complex challenge. The primary therapeutic strategy for severe burn patients involving greater than 20–25% total body surface area (TBSA) is early aggressive intravenous (IV) fluid resuscitation to replace lost volumes, improve tissue oxygenation, and avoid burn shock.^1^ Some guidelines recommend up to 20 L of IV fluid within the first 24 h.^2^ However, aggressive resuscitation is now known to aggravate burn trauma by failing to correct cardiac and lung dysfunction and prevent endotheliopathy, capillary leakage syndrome and other systemic derangements, that occur within the first 48 h after burn injury.^3–6^ Emerging therapeutics that target pulmonary, cardiovascular, endothelial-glycocalyx and organ dysfunction in the management of burn shock are gaining recognition.^7–9^

Over the past decade, we have been developing a small-volume IV fluid resuscitation therapy comprising adenosine, lidocaine and magnesium (ALM) for hemorrhagic shock, traumatic brain injury, sepsis, endotoxemia, and surgical trauma.^10–17^ ALM therapy comprises a small-volume bolus for point-of-injury and prehospital care for resuscitation and a low-volume drip for stabilization during continuum-of-care.^14,17^ ALM therapy has been reported to protect the endothelium following exsanguinating trauma,^18^ and to reduce microvascular dysfunction and protect against acute lung injury following sepsis in rats and pigs.^10,12,13^ Importantly, the ALM resuscitative and whole body protective effects are not conferred using adenosine, lidocaine or magnesium alone; only by the combination of actives.^14,17,19,20^ The aim of the present study was to investigate the early protective effects of small-volume ALM fluid resuscitation in a rat model of 30% TBSA thermal injury. We hypothesize that ALM therapy will protect against loss of lung alveolar-capillary membrane integrity, support cardiovascular and endothelial function, and reduce multiple organ dysfunction following burn trauma.

## METHODS

This study was approved by the Institutional Animal Ethics Committee (Approval Number: A2479) and conforms to the National Health and Medical Research Council Australian Code for the Care and Use of Animals for Scientific Purposes, 8th Edition, 2013, and complies with the Queensland Animal Care and Protection Act, 2001 (Act No.64 of 2001), and institutional guidelines. Conventional adult male Sprague-Dawley rats (320–340 g) obtained from the university’s breeding colony, were housed in a 14–10-hr dark–light cycle with free access to food and water *ad libitum*. Anesthesia was induced with 5% isoflurane/100% O_2_ and maintained with continuous 0.5–1.5% isoflurane/100% O_2_. Animals received 0.05 mg/kg Buprenorphine Hydrochloride (Temgesic) subcutaneously for pain relief prior to recovery from anesthesia.

### Animal Preparation and Surgical Instrumentation

**Figure 1.**
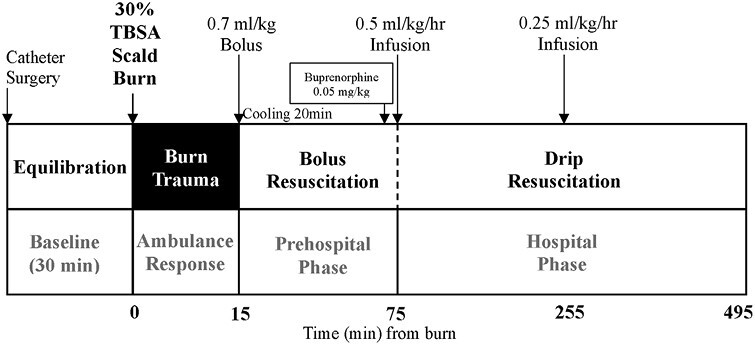
Schematic of Study Protocol. Intervals of the protocol are based on the clinical environment after burn injury including ambulance response time, and both prehospital and hospital resuscitation phases.

Aseptic technique was used for all animal handling and surgical instrumentation. Under anesthesia, the dorsal and lateral surfaces were shaved, disinfected with povidone-iodine solution, and cleaned with 70% ethyl alcohol. Core body temperature was monitored using a rectal probe (T-type Pod, ADInstruments, Bella Vista, Australia) and maintained between 35.5 and 37.5°C with a homeothermic blanket (Physitemp, ADInstruments). The technique for femoral vascular access has previously been described.^15,21^ Sterile chronic catheters (Access Technologies, Skokie, IL, USA) were inserted in the left femoral artery and vein and attached to an Instech dual access vascular port fitted with a jacket (Walker Scientific, Western Australia). Venous access allowed for fluid infusion, and arterial access allowed for conscious blood sampling and continuous blood pressure (BP) monitoring (BridgeAmp/PowerLab; ADInstruments). Subcutaneous electrocardiogram (ECG) leads were attached in Lead II configuration. After instrumentation, animals remained anesthetized for a 30-min stabilization period prior to recording baseline measurements (Figure 1).

Animals (*n* = 25) were randomly assigned to: 1) Sham (surgical instrumentation, without burn, *n* = 5), 2) Saline group (*n* = 10), or 3) ALM group (*n* = 10). The validated 30% TBSA full-thickness thermal burn methodology has previously been described.^22^ TBSA and total surface area burnt was calculated using the Meeh formula (TBSA = kW^2/3^), where *k* = 9.83, and W = weight (kg).^23^ A cradle with a prefabricated aperture of 68 cm^2^ (5 × 13.6 cm) was created based on the selected 320–340 g weight range. With the left lateral dorsal side exposed, the cradle was immersed into a 96 °C water bath for 8-sec, followed immediately by exposure of the right lateral dorsal side for 8-sec, avoiding the paws, tail and head. This protocol has previously been demonstrated to produce a full-thickness burn injury with metabolic response.^22^ Sham animals received anesthesia and surgical instrumentation, and were exposed to room temperature (RT) water.

Following burn induction, the dorsal surface was dried gently with clean paper towel and the anesthetized animal placed prone on a clean, dry sterile underpad over a 37 °C homeothermic blanket to maintain normothermia, and continuous hemodynamic monitoring recommenced. 15-min after induction of burn injury a 0.7 ml/kg bolus of 1) 3% NaCl (Sham and Saline group) or 2) 3% NaCl ALM (ALM group) was administered at ~0.06 mL/s (Bolus Resuscitation) (Figure 1). ALM bolus comprised 3% NaCl with 1 mM Adenosine, 3 mM Lidocaine and 2.5 mM MgSO_4_, as per previous trauma studies.^11,16,21^ Simultaneously, the burn was cooled with RT water spray continuously for 20-min, after which the burn site was covered with 3M Tegaderm Film (Medshop, Australia).

Animals were recovered from anesthesia 75-min after injury and commenced a low-volume resuscitation infusion of 0.5 mL/kg/h 0.9% NaCl (Sham and Saline group) or 0.9% NaCl ALM (ALM group; 50 mg Adenosine, 100 mg Lidocaine, 50 mg MgSO_4_/10 mL 0.9% NaCl) (Drip Resuscitation) (Figure 1). At 255-min post-burn the infusion rate was reduced to 0.25 mL/kg/h until 495-min. A homeothermic blanket (37°C) was placed under the recovery cage to maintain warmth. Animals were euthanized at 495-min post-burn with intraperitoneal injection of 100 mg/kg pentobarbitone sodium (Lethabarb) for tissue sampling.

### Hemodynamic Measurements

Hemodynamics (mean arterial pressure [MAP], systolic pressure [SP], diastolic pressure [DP], heart rate [HR]) and core body temperature were continuously monitored and recorded for the first 75-min after burn. After anesthesia recovery MAP, SP and DP were continuously measured via the femoral artery catheter and body temperature was recorded hourly using a noninvasive Infrared Digital Thermometer. Cardiac function was determined using transthoracic echocardiography as previously described.^16,21,24^

### Blood Chemistry and Hematology

Blood was sampled at baseline, 75-min, 255-min, and 495-min for blood chemistry (Radiometer ABL800, Radiometer Pacific, Victoria), and complete blood count (VetScan HM5, REM Systems, New South Wales). Oxygen delivery (OD) was calculated from: OD (ml O_2_/min) = CO(ml/min) x arterial oxygen content (CaO_2_; ml O_2_/100 mL), where CaO_2_ = (Hgb x 1.36 x sO_2_) + (0.0031 x pO_2_).^25^

### Endothelial Injury and Inflammation

Plasma samples from 75-min, 255-min, and 495-min were analyzed for soluble intercellular adhesion molecule-1 (sICAM-1), soluble E-selectin (sE-selectin), and Adiponectin, using Milliplex Rat Vascular Injury Magnetic Bead Panel 2 (Lot: 3070571). sICAM-1 and sE-selectin are biomarkers of endothelial injury,^26^ and have previously been shown to be elevated following burn injury.^27,28^ Plasma samples were also analyzed for inflammatory markers interleukin (IL)-1α, IL-1β, IL-2, IL-4, IL-6, IL-10, IL-12/p70, IL-13, Macrophage Inflammatory Protein (MIP)-1α, tumor necrosis factor (TNF)-α, Interferon (IFN)-γ, Monocyte Chemoattractant Protein (MCP)-1, and Regulation on Activation, Normal T Cell Expressed and Secreted (RANTES), using Milliplex Rat Cytokine/Chemokine Magnetic Bead Panel (Lot: 294299). Milliplex assays (Abacus ALS, Meadowbrook, Queensland) were analyzed using Magpix 200 analyzer (Luminex Corporation, Austin, Texas, USA) according to manufacturer’s instructions. Plasma samples from, baseline, 75 min, 255 min, and 495 min post-burn were also analyzed for serum protein alpha1-acid glycoprotein using Rat Alpha 1 Acid Glycoprotein/AGP ELISA Kit (ab157729, Abcam, Melbourne, Australia). Lung, heart and gut tissues were assayed for malondialdehyde (MDA; Lipid peroxidation kit, Sigma Aldrich, Australia) to quantify lipid peroxidation.

### Histological Analysis

Formalin-fixed gut (jejunum), heart, and lung samples were processed using the Leica Histocore Pearl tissue processor, paraffin-embedded using the Histocore Arcadia embedding center, sectioned using the Leica RM2255 automated rotary microtome, stained with hematoxylin and eosin (H&E), and examined using light microscopy (Olympus CKX41 Nikon Digital Sight). Lung sections were evaluated for leukocyte infiltration and pulmonary congestion, and heart sections were scored based on edema, congestion, inflammation, and hemorrhage.^21,29,30^ Intestinal morphology and inflammatory response was assessed with the standardized Chiu scoring system.^31^ Jejunum was selected for histopathological examination since it is the first area of the intestine to show damage post-burn.^32^ For each tissue, three sections were scored by two independent observers blinded to experimental groups using coded slides.

### Statistical Analysis

*A priori* power analysis was conducted using G-power^3^ program to determine sample size to minimize Type 1 errors (Cohen’s d effect size = 1.4^11^; α = 0.05; Sample size = 10 per treatment group; Power (1-β) = 0.84). The sample size for the Sham group, which was included to determine the effect of anesthesia and catheterization surgery in the absence of burn injury, was based on previous studies.^33^ SPSS Statistical Package 24 (IBM) was used for statistical analysis. All values are expressed as mean ± SEM. Data normality was assessed with Shapiro–Wilks test. Nonparametric data were evaluated using Kruskal–Wallis. Analysis of variance (ANOVA) was used to evaluate parametric data with Tukey’s HSD or Dunnett’s post-hoc test dependent on Levene’s homogeneity of variance. Longitudinal data was analyzed using General Linear Model Repeated Measures ANOVA with Greenhouse-Geisser correction if Mauchy’s Test of Sphericity is not met. MDA assays were analyzed using Prism 8 4-parameter-logistic curve fitting (GraphPad), and Analyst 5.1 software was used for analysis of Milliplex assays. Statistical significance was defined as *P* < .05.

## RESULTS

### Survival and Hemodynamics

**Figure 2.**
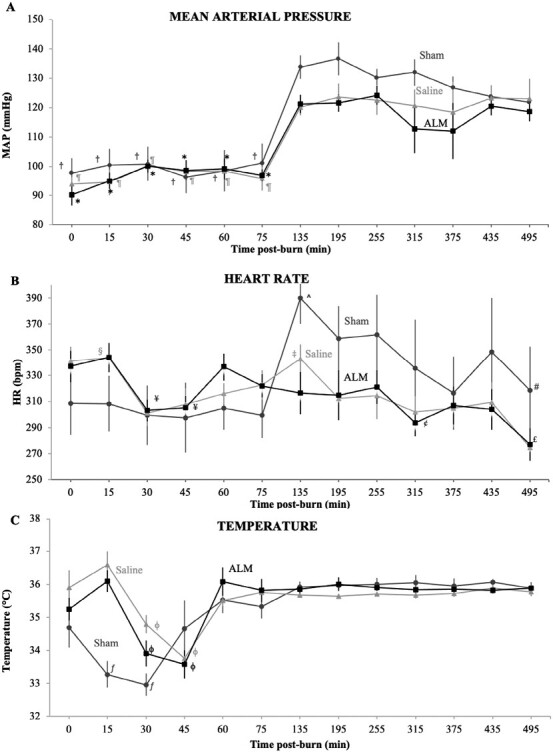
Mean arterial blood pressure (MAP, mmHg) (A), heart rate (HR; bpm) (B), and temperature (ºC) (C), in Shams, Saline Controls and ALM-treated animals after 30% TBSA thermal burn. Hemodynamics were measured at 15-min intervals for the first 75-min post-burn, and then hourly thereafter. Data presented as mean±SEM. ^†^*P* < .05 compared to 75-495 min; ^¶^*P* < .05 compared to 75-255 min and 375-435 min; * *P* < .05 compared to 75-195 min and 375-435 min; ^^^*P* < .05 compared to 45-60 min, 375 min and 495 min; ^#^*P* < .05 compared to 435 min; ^§^*P* < .05 compared to 30-75 min; ^‡^*P* < .05 compared to 30-75 min and 495 min; ^¥^*P* < .05 compared to baseline and 15 min; ^£^*P* < .05 compared baseline, 30 min, 60-255 min, and 375-435; ^¢^*P* < .05 compared to baseline, 15 min, 60-75 min, and 195 min; ^ƒ^*P* < .05 compared to baseline and 60-495 min; ^ϕ^*P* < .05 compared to baseline, 15 min, and 60-495 min.

**Figure 3.**
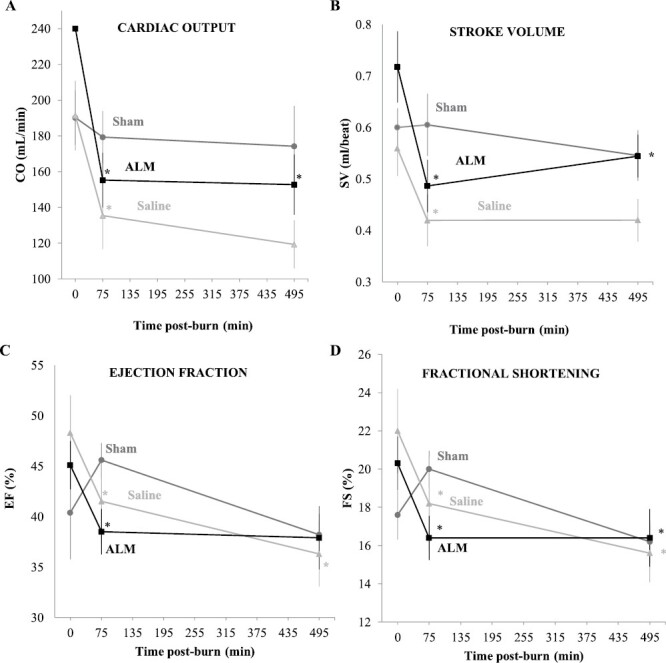
Cardiac output (CO, ml/min) (A), stroke volume (SV, ml/beat) (B), ejection fraction (EF, %) (C), and fractional shortening (FS, %) (D) in Shams, Saline Controls and ALM-treated animals at baseline, and 75 min and 495 min after 30% TBSA thermal burn. Data presented as mean ±SEM. * p<0.05 compared to baseline.

Survival was 100% for Shams and 90% for both ALM and Saline groups. Both deaths were due to burn shock defined by MAP<25mmHg for 5min (Time of death: Saline 395-min; ALM 360-min). 3% NaCl±ALM bolus increased MAP from 95-100mmHg in both Saline controls and ALM group, while HR fell by ~12% from ~340-300bpm in both groups after burn and bolus administration (Fig 2A,B). ALM attenuated the significant rise in HR compared to Sham and Saline animals at 135-min following anesthesia recovery, and maintained a stable HR during 0.9% NaCl ALM drip infusion. Cardiac function was significantly reduced in both burn groups from baseline to 75-min post-burn, with no group differences (Fig 3). ALM increased stroke volume (SV) at 495-min, and improved O_2_ delivery by 21% compared to Saline group (2726 ± 386 vs. 2163 ± 255 ml O_2_/min; p=0.418). Burn injury was associated with an ~1°C increase in body temperature (Fig 2C). Burn cooling from 15-35min significantly reduced temperature to 33-34°C, however from 60-min post-burn all groups maintained normothermia (Fig 2C).

### Organ Injury

**Figure 4.**
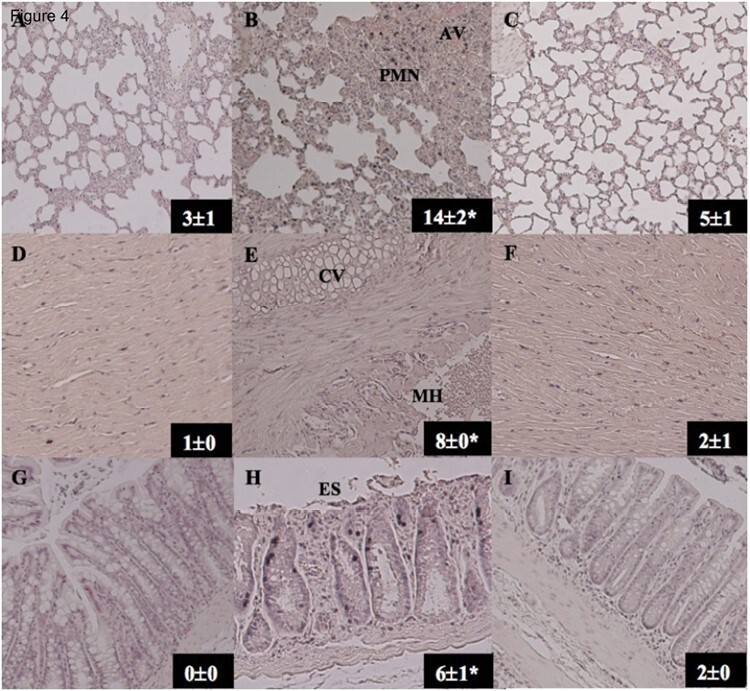
Representative histological images (x40) from lung, heart, and gut (jejunum) in Shams (A, D, G), Saline controls (B, E, H), and ALM group (C, F, I) after 30% TBSA thermal burn. Injury scores presented for each tissue are the average of two independent blinded investigators (mean±SEM). PMN = polymorphonuclear cell infiltration; AV = alveolar hemorrhage; CV = cytoplasmic vacuolation, indicated by large area of clearly defined white vacuoles; MH = myocardial hyperemia; ES = epithelial shedding, indicated by remnants of epithelial cells in the intestinal lumen. *P < .05 compared to Sham and ALM group.

Histological injury scores for lung, heart and gut were significantly higher in Saline controls compared to Shams and ALM group (Fig 4). ALM-treated lungs had minimal destruction of alveoli compared to Saline animals, which also showed alveolar hemorrhage and inflammatory cell infiltration (Fig 4B-C). Cardiac tissue in ALM group was comparable to Shams, whereas Saline group had evidence of hyperemia and cytoplasmic vacuolation (Fig 4D-F). Cytoplasmic swelling was also present in Saline group gut, as well as extensive epithelial shedding (Fig 4H).

**Figure 5.**
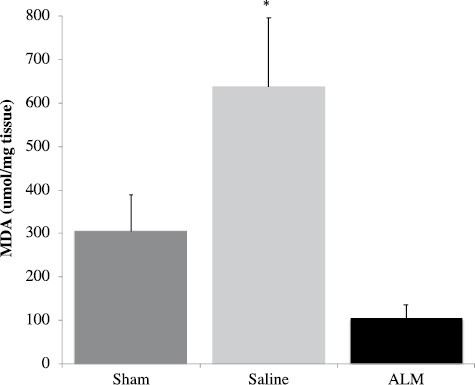
Lung malondialdehyde concentration in Shams, Saline controls, and ALM-treated animals 495-min after 30% TBSA thermal burn. Data presented as mean±SEM (μM/mg tissue). * *P* = .023 compared to ALM group.

ALM therapy significantly reduced MDA levels in the lungs compared to Saline (p=0.023; Fig 5). MDA levels did not differ between groups in heart (p=0.242) or gut tissue (p=0.782).

### Inflammation and Endothelial Injury

**Figure 6.**
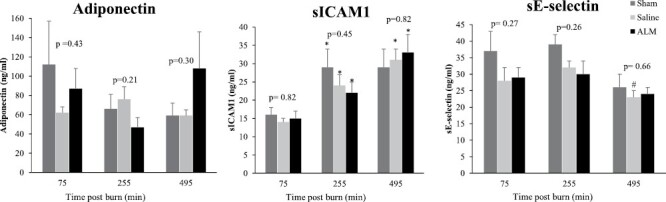
Adiponectin (A), soluble intercellular adhesion molecule-1 (sICAM1; B), and sE-selectin (C) after 30% TBSA thermal burn. Data represent mean±SEM. *P*-value represents between-groups effect. * *P* < .05 compared to 75-min; ^#^*P* < .05 compared to 255-min.

There were no significant differences in sICAM-1, or soluble E-Selectin between groups at any time-point after burn injury (Fig 6B-C). sICAM-1 increased significantly from 75-min to 255-min and 495-min in all groups, including Shams (*P* < .05) (Fig 6B). Adiponectin showed a 230% increase in the ALM group at 495-min, however this change was not statistically significant (Fig 6A). Thermal injury was not associated with systemic inflammation in the first 495-min after burn with no differences between Sham animals and Saline controls or ALM-treated animals across 13 inflammatory markers (data not shown).

Total leukocytes increased significantly in Saline controls from 10.4 ± 1.2 to 16.3 ± 0.9 x10^9^/L at 75-min post-burn (*P* < .05 vs Sham and baseline), whereas there was no change in ALM-treated rats (Table 1). Neutrophil (%) increased significantly in all groups, including Shams, from baseline to 495-min, with no differences between groups (p=0.062). Total red cells, hemoglobin, and hematocrit were all significantly increased in both burn groups compared to Shams 75-min post-burn (Table 1). Plateletcrit was significantly higher in Saline controls at 495-min compared to Shams and ALM group. Lactate did not differ between Sham animals or burn groups; however base excess was significantly lower in Saline and ALM groups 495-min post-burn (p=0.003) (Table 2). Burn groups both became significantly acidotic after 255-min (*P* < .001 vs Sham), and pH was 7.21 in both burn groups at 495-min (*P* = .002 vs Sham).

**Table 1.** Complete blood count at baseline and 75 and 495-min post-burn

Marker	Time	Sham	Saline	ALM	*P*-value
WBC (10^9^/L)	Baseline	11.7 ± 2.5	10.4 ± 1.2	9.3 ± 0.9	.530
	75 min	10.8 ± 1.5	16.3 ± 0.9^‡^	13.2 ± 1.4	.029
	495 min	10.8 ± 2.1	15.5 ± 1.1^#^	12.9 ± 1.1	.089
LYM (%)	Baseline	73 ± 5	73 ± 3	73 ± 3	.999
	75 min	55 ± 6^#^	57 ± 3^#^	60 ± 2^#^	.552
	495 min	30 ± 4^¶^	40 ± 3^¶^	44 ± 3^¶^	.028
NEU (%)	Baseline	22 ± 5	23 ± 2	21 ± 3	.590
	75 min	39 ± 7^#^	37 ± 3^#^	32 ± 2^#^	.442
	495 min	61 ± 3^¶^	53 ± 3^¶^	49 ± 3^¶^	.062
MON (%)	Baseline	5 ± 2	4 ± 1	6 ± 1	.641
	75 min	6 ± 1	5 ± 1	7 ± 1	.366
	495 min	8 ± 1	7 ± 1	6 ± 1	.178
RBC (10^12^/L)	Baseline	9.13 ± 0.36	9.37 ± 0.12	9.19 ± 0.09	.563
	75 min	8.64 ± 0.18	10.01 ± 0.09^†^	9.68 ± 0.21^†^	<.001
	495 min	7.10 ± 0.41^¶^	9.35 ± 0.26	9.02 ± 0.21	<.001
HgB (pg/ml)	Baseline	15.6 ± 0.4	16.9 ± 0.2^¥^	16.3 ± 0.2	.019
	75 min	14.9 ± 0.3	18.0 ± 0.2^†^	18.0 ± 0.3^†^	<.001
	495 min	12.3 ± 0.7^¶^	16.7 ± 0.5^¥^	16.3 ± 0.3^¥^	<.001
HCT (%)	Baseline	49 ± 1	52 ± 1	51 ± 1	.198
	75 min	47 ± 1	53 ± 0^¥^	53 ± 1^¥^	<.001
	495 min	37 ± 3^¶^	49 ± 1^ϕ^	48 ± 1^ƒ^	<.001
PCT (%)	Baseline	0.19 ± 0.04	0.22 ± 0.03	0.21 ± 0.02	.862
	75 min	0.23 ± 0.02	0.24 ± 0.03	0.25 ± 0.02	.873
	495 min	0.14 ± 0.01^^^	0.21 ± 0.02^§^	0.17 ± 0.01^ϕ^	.009

Data represent mean±SEM. *P*-value represents between-groups effect. WBC = white blood cell; LYM = lymphocyte; NEU = neutrophil; MON = monocyte; RBC = red blood cell; HgB = hemoglobin; HCT = hematocrit; PCT = plateletcrit. ^#^*P* < .05 compared to baseline; ^‡^*P* < .05 compared to Sham and baseline; ^ϕ^*P* < .05 compared to Sham and 75 min; ^¶^*P* < .05 compared baseline and 75min; ^†^*P* < .05 compared to Sham, baseline and 495min; ^¥^*P* < .05 compared to Sham; ^ƒ^*P* < .05 compared to Sham, baseline and 75 min; ^^^*P* < .05 compared to 75 min; ^§^*P* <.05 compared to Sham and ALM.

**Table 2.** Blood chemistry at baseline and 75 and 495-min post-burn

Analyte	Time	Sham	Saline	ALM	*P*-value
Lactate (mmol/L)	Baseline	1.18 ± 0.06	1.18 ± 0.08	1.28 ± 0.10	.650
	75 min	1.32 ± 0.19	1.44 ± 0.19	1.64 ± 0.15^#^	.491
	255 min	1.46 ± 0.19	1.42 ± 0.20	1.93 ± 0.26^#^	.629
	495 min	1.36 ± 0.17	1.54 ± 0.28	1.62 ± 0.35	.950
Base excess (mmol/L)	Baseline	4.22 ± 0.51	3.13 ± 0.43	2.78 ± 0.39	.140
	75 min	1.64 ± 0.54	−1.31 ± 0.60^‡^	−1.97 ± 0.90^#^	.024
	255 min	-0.78 ± 1.72^#^	−7.47 ± 0.89^ψ^	−6.41 ± 2.46^#^	.099
	495 min	0.40 ± 1.85	−10.53 ± 1.72^Ω^	−11.15 ± 2.10^Ω^	.003
pH	Baseline	7.34 ± 0.05	7.28 ± 0.04	7.23 ± 0.04	.260
	75 min	7.22 ± 0.03	7.24 ± 0.04	7.26 ± 0.02	.651
	255 min	7.35 ± 0.03^ϕ^	7.23 ± 0.01^*^	7.20 ± 0.02^+^	<.001
	495 min	7.38 ± 0.03^^^	7.21 ± 0.03^*^	7.21 ± 0.02^*^	.002
Potassium (mmol/L)	Baseline	5.32 ± 0.15	4.61 ± 0.16^*^	4.58 ± 0.10^*^	.006
	75 min	4.62 ± 0.12^#^	5.48 ± 0.27^#^	5.47 ± 0.27^#^	.114
	255 min	4.42 ± 0.09^#^	5.35 ± 0.21^‡^	5.65 ± 0.20^‡^	.003
	495 min	4.48 ± 0.23^#^	5.86 ± 0.37^#^	6.43 ± 0.37 ^Ω^	.012

Data presented as mean ± SEM. *P*-value represents between-groups effect. HCO_3_^−^ = bicarbonate. Baseline bicarbonate values not available due to oximetry measuring error. ^#^*P* < .05 compared to baseline; ^‡^*P* < .05 compared to Sham and baseline; ^ψ^*P* < .05 compared to baseline and 75 min; ^Ω^*P* < .05 compared to Sham, baseline, 75 min and 255 min; ^ϕ^*P* < 0.05 compared to 75 min; ^^^*P* < .05 compared to 75 and 255 min; * *P* < .05 compared to Sham; ^+^*P* < .05 compared to Sham and 75 min; ^¶^*P* < .0.05 compared to 255 min; ^ƒ^*P* < .0.05 compared to Sham, 75 and 255 min.

## DISCUSSION

Combating early mortality and organ failure from burn shock urgently requires new therapies to restore homeostasis.^34–36^ This is the first study to examine small-volume ALM therapy in a preclinical model of severe burns. Using early ALM bolus and infusion, we showed that: 1) Shams underwent significant changes to physiological, immune and hematological status over time; 2) ALM significantly reduced oxidative stress in the lung and preserved alveolar integrity compared to Saline controls and Shams; 3) ALM increased cardiac function and tissue O_2_ supply by ~20% compared to Saline controls, however, the increases were not significant; 4) Circulating neutrophils and sICAM-1 increased 2.3-2.8-fold and 1.5-2-fold, respectively, in all three groups, which is consistent with widespread endothelial activation; 5) ALM animals had significantly increased adiponectin values at 495-min; and 6) the acute phase plasma protein AGP significantly increased 1.6-times in Saline and ALM treatment groups. We will now discuss these major findings.

### Shams: An Often Neglected Variable in Animal Research

An overlooked factor in animal research is the effect of anesthesia and preparatory surgery on the physiological state of the animal over the course of the experiment separate from experimental trauma and administering a treatment.^15,37^ In the study, Sham animals underwent surgical instrumentation and received saline infusion without burn trauma. We showed MAP and HR in Shams were up to 35% higher early post-anesthesia compared to burn trauma plus Saline or ALM treatment, which dampened these early effects (Fig 2AB). Shams also showed high MDA levels in lung tissue (Fig 5), indicating alveolar damage from lipid peroxidation, and high adiponectin levels (Fig 6), perhaps to counter this effect. Immune function was also activated by anesthesia and preparatory surgery in our study. We report nearly a two-fold increase in circulating neutrophils at 75-min and high levels of sICAM-1 which is consistent with histopathology, endothelial and lung injury (Table 1, Fig 6B, Fig 4A). Similarly, the hematological profile of Sham animals changed significantly over time with 22-24% falls in total red cells, hemoglobin, and hematocrit at 495-min compared to baseline values (Table 1). Overall, these data demonstrate that Shams are not inert or passive groups, and play significant roles in altering physiological and immune status in the absence of the primary trauma (i.e., burn) or following drug treatment being investigated. Shams should be viewed as models of trauma themselves albeit less severe.

### ALM Protected Against Oxidative Stress and Ischemic Injury in Lungs and Gut

**Figure 7.**
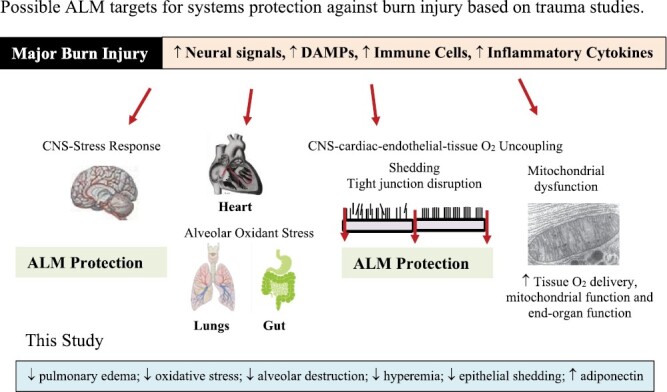
Diagram showing how ALM therapy may blunt the CNS sympathetic hyperdrive stress response following major burn injury with multiorgan protection. ALM: adenosine, lidocaine and magnesium; DAMP: damage-associated molecular pattern; CNS: central nervous system.

A major finding of the present study was that ALM therapy significantly reduced lung injury scores and protected the lungs from oxidative stress following severe burns, as measured by levels of malondialdehyde, an index of lipid peroxidation and free radical generation (Fig 5). Reactive oxygen species (ROS) are key mediators of acute lung injury (ALI) and acute respiratory distress syndrome (ARDS),^38^ and are common complications in burns patients that are associated with increased mortality.^39–41^ Improved alveolar protection was also supported by histological scores which indicated reduced local alveolar hemorrhage and inflammatory cell infiltration compared to Saline controls or Shams (Fig 4A-C). At present, we don’t know the mechanisms of ALM protection of the lung but it may relate to endothelial protection and decreased endothelial-glycocalyx shedding, as we, and others, have been shown following hemorrhagic shock in the rat (Fig 7).^42–44^ The higher abundance of glycocalyx in lungs compared to heart and gut,^8^ may be one reason why we found the greatest protective response in lungs.

Gut histopathological injury scores were lower after ALM treatment compared to Saline controls, with significant reductions in epithelial shedding and cytoplasmic swelling (Fig 3H). After severe burns, intestinal barrier dysfunction develops early,^45^ and has been associated with shock, bacterial translocation, systemic inflammation, multiple organ dysfunction, and sepsis (Fig 7).^46,47^ In rodents after 30% TBSA burn, increased intestinal permeability peaks at ~6-hr,^45,46^ and is believed to be due to sympathetic-mediated vasoconstriction leading to ischemia and damage.^48^ Since resuscitation volume and fluid type are increasingly recognized to directly impact intestinal barrier function,^49,50^ small-volume ALM therapy’s protection warrants further investigation.

### ALM Improved Cardiac Output and O_2_ Delivery

In our study, ALM therapy reduced burn-induced cardiac depression compared to Saline controls from 75-min to 495-min post-burn, with greater stabilization of CO, SV, ejection fraction, and fractional shortening (Fig 3). However the increases were not statistically significant. Depressed cardiac contractility has been reported to occur as early as 15-min post-burn injury, *before* the reduction in plasma volume^51^ and large aggressive volumes of standard-of-care crystalloid IV fluids do not ameliorate early burn-associated cardiac depression.^4^ We further showed that the ~20% increase in pump function in ALM-treated animals led a similar increase in oxygen delivery at 495-min post-burn, which is consistent with our previous ALM resuscitation studies on pig trauma.^12^ ALM’s cardioprotective effects were supported histologically by significantly less hyperemia and vacuolization of myocytes (Fig 4D-F). These data may be clinically significant because that are no current therapies to prevent left ventricular contractile dysfunction after severe burns.

### ALM Therapy Was Partially Protective Against Microvascular and Endothelial Damage

ALM therapy did not result in a significant reduction in circulating vascular injury markers in the first ~8-hr after burn injury (Fig 6). However, ALM treatment did partially prevent hemoconcentration and maintain a significantly lower plateletcrit compared to Saline controls, which are clinical indicators of endothelial and microvascular dysfunction and inflammation in humans (Table 1).^52^ An interesting finding of the present study was that ALM therapy increased plasma adiponectin 2.3-times relative to Saline controls and Shams between 255 and 495-min. This is contrary to 55% decreases found in burn patients,^53^ and after other traumatic injuries.^53–56^ We have similarly shown ALM therapy increases plasma adiponectin 1.5-times after hemorrhagic shock and traumatic brain injury (unpublished data). At present, we do not know the underlying mechanisms for increases in circulating adiponectin but they may reflect decreased post-burn oxidative stress and maintainance of endothelial integrity.^54,57^ Increased adiponectin may also have potential long-term benefits following burn injury, through regulation of cutaneous wound healing.^58^

Additional studies are required to test these hypotheses and to further understand the underlying mechanism of action of ALM’s potential benefits post-burn. In other trauma models we have identified the importance of sympathetic stress response and switch to parasympathetic dominance for maintenance of cardiovascular coupling to protect the endothelium, maintain mitochondrial energetics, and minimize immune dysregulation, inflammation, coagulopathy and multiple organ dysfunction (Fig 7).^21,44,59^

### Increased Plasma Alpha 1-Acid Glycoprotein (AGP) and Implications to ALM’s Therapeutic Effect

Another interesting finding was that acute phase protein AGP significantly increased 1.6-fold over 495-min after burn trauma (*P* < .001) (Supplementary Content 1). Unfortunately we did not measure AGP in Shams to assess the impact of surgical trauma in the absence of burn trauma. As lidocaine is known to strongly bind to plasma AGP (80%),^60^ it is possible that increased AGP reduced lidocaine’s bioavailability and is partly responsible for ALM’s reduced therapeutic effect. To explore this further, we undertook a post-hoc analysis to examine changes in plasma AGP level (Supplementary Content 1) in rat burn and noncompressible hemorrhagic shock models and found that compared to the burns model, AGP was significantly lower (p=0.019) at a similar time-point in the hemorrhagic shock model, suggesting reduced bioavailbility of lidocaine in ALM-treated burn animals. It is possible therefore that early increases in plasma AGP post-burn may have reduced ALM’s therapeutic efficacy compared to other trauma models, and warrants further studies with higher doses.

### Limitations of the Model, Buprenorphine Analgesia, and Future Studies

In our burn model we found that systemic inflammation was not activated in the first ~8 hrs, with no differences in cytokine or chemokine levels compared to Shams. This has been reported before in other rodent burn models.^61,62^ Significant alterations in cytokines are not found until after 24-hr,^63^ and in humans IL-6 and IL-10 do not peak until 24-48-hr.^64^ These data demonstrate that longer monitoring times are required in our rodent model before systemic inflammatory processes are activated. Another limitation of the present study is the use of the opioid buprenorphine as an analgesic. Guillory and colleagues have shown that buprenorphine alters the hemodynamic response to injury and may not be an appropriate choice for a model of severe burn injury.^65^ We have subsequently shown that ALM in rat and pig models of hemorrhagic shock are contraindicated in the presence of buprenorphine,^66,67^ and we have switched to nonsteroidal anti-inflammatory drug (NSAID) Carprieve® (carprofen) analgesia. The use of buprenorphine may be one reason for ALM’s reduced efficacy in burn compared to hemorrhagic shock and other trauma models. In addition, the significant increase in the plasma lidocaine-binding binding protein AGP necessitates investigating higher doses of ALM fluid therapy, which may amplify its protective effects following severe burns.

## CONCLUSIONS

In conclusion, treatment of severe burns with small-volume ALM in the immediate post-burn period showed significant lung protection and improved cardiac and gut protection. Further studies examining increasing doses of ALM with long-term follow-up are required to determine the potential protective effect of ALM in severe burns. Lastly, our results further demonstrate that Sham animals are not inert or passive groups and should be viewed as models of trauma themselves albeit less severe.

## Supplementary Material

irad127_suppl_Supplementary_Data
