# Supplementary material for: Lung Protection After Severe Thermal Burns With Adenosine, Lidocaine, and Magnesium (ALM) Resuscitation and Importance of Shams in a Rat Model
Source: J Burn Care Res. 2023 Aug 21;45(1):216–26. doi: 10.1093/jbcr/irad127 (PMC10768784; doi:10.1093/jbcr/irad127)
Supplement: irad127_suppl_Supplementary_Data [file irad127_suppl_supplementary_data.pdf]

Supplementary Content 1

Method

Plasma sampled from, baseline, 75 min, 255 min, and 495 min post-burn were analyzed for alpha-1-acid glycoprotein using Rat Alpha 1 Acid Glycoprotein/AGP ELISA Kit (ab157729, Abcam, Melbourne, Australia).

Result

A post-hoc analysis showed that AGP significantly increased during burn trauma. Compared to previous study of ALM in uncontrolled hemorrhage, at a similar time, burn trauma evoked a significantly higher AGP response. (19.82 ± 0.65 µg/ml vs. 16.90 ±0.46 µg/ml; p=0.019).

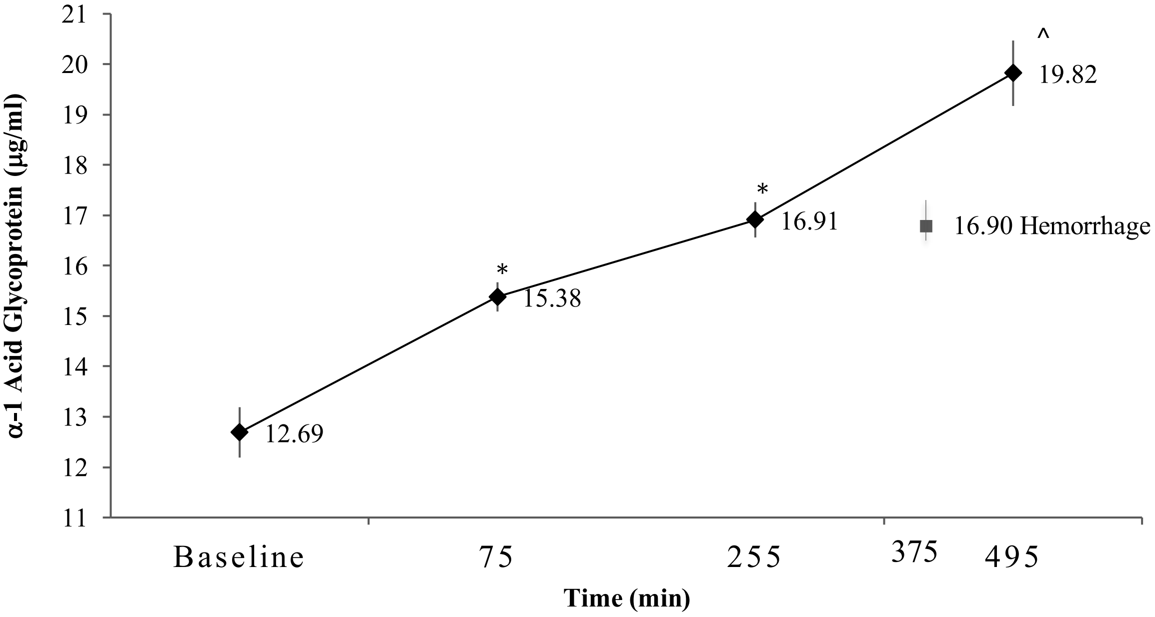

Plasma samples from 75-min, 255-min, and 495-min in the burn study and at 375-min from an uncontrolled hemorrhage study were analyzed of AGP. Data presented as mean±SEM (µg/ml). * p<0.05 vs. Baseline; ^ p<0.05 vs. Baseline and 75-min and 255-min.
